# Pore texture analysis in automated 3D breast ultrasound images for implanted lightweight hernia mesh identification: a preliminary study

**DOI:** 10.1186/s12938-021-00859-7

**Published:** 2021-02-25

**Authors:** Jiting Yang, Haiyan Li, Jun Wu, Liang Sun, Dan Xu, Yuanyuan Wang, Yufeng Zhang, Yue Chen, Lin Chen

**Affiliations:** 1 Department of Electronic Engineering, Yunnan University, Kunming, China; 2 Department of Gastrointestinal and Hernia Surgery, The First Affiliated Hospital of Kunming Medical University, Kunming, China; 3 Department of Electronic Engineering, Fudan University, Shanghai, China; 4 Department of Ultrasound, Huadong Hospital, Fudan University, Shanghai, China

**Keywords:** ABUS, Abdominal wall hernia, LW mesh, Implanted mesh identification, Pore texture analysis

## Abstract

**Background:**

Precise visualization of meshes and their position would greatly aid in mesh shrinkage evaluation, hernia recurrence risk assessment, and the preoperative planning of salvage repair. Lightweight (LW) meshes are able to preserve abdominal wall compliance by generating less post-implantation fibrosis and rigidity. However, conventional 3D imaging techniques such as computed tomography (CT) and magnetic resonance imaging (MRI) cannot visualize the LW meshes. Patients sometimes have to undergo a second-look operation for visualizing the mesh implants. The goal of this work is to investigate the potential advantages of Automated 3D breast ultrasound (ABUS) pore texture analysis for implanted LW hernia mesh identification.

**Methods:**

In vitro, the appearances of four different flat meshes in both ABUS and 2D hand-held ultrasound (HHUS) images were evaluated and compared. In vivo, pore texture patterns of 87 hernia regions were analyzed both in ABUS images and their corresponding HHUS images.

**Results:**

In vitro studies, the imaging results of ABUS for implanted LW meshes are much more visualized and effective in comparison to HHUS. In vivo, the inter-class distance of 40 texture features was calculated. The texture features of 2D sectional plans (axial and sagittal plane) have no significant contribution to implanted LW mesh identification. Significant contribution was observed in coronal plane. However, since the mesh may have spatial variation such as shrinkage after implantation surgery, the inter-class distance of 3D coronal plane pore texture features are bigger than 2D coronal plane, so the contribution of 3D coronal plane pore texture features are more valuable than 2D coronal plane for implanted LW mesh identification. The use of 3D pore texture features significantly improved the robustness of the identification method in distinguishing between LW mesh and fascia.

**Conclusions:**

An innovative new ABUS provides additional pore texture visualization, by separating the LW mesh from the fascia tissues. Therefore, ABUS has the potential to provides more accurate features to characterize pore texture patterns, and ultimately provide more accurate measures for implanted LW mesh identification.

## Background

Repair of abdominal wall hernias is one of the most frequently performed operations across the world [1–3]. It has been estimated that 250,000 ventral hernia repairs are being done in the USA yearly [4, 5]. Today, most abdominal wall hernias are treated by placement of mesh to repair the abdominal wall defect [6–8]. Several reports have shown that compared with simple sutures, mesh is superior, with significantly reduced recurrence rates [8–11]. However, implanted mesh is a foreign body and is subject to a variety of complications, with recurrence remaining an unsolved problem and reoperation increasingly common [6, 12]. Therefore, sonologists now need perform an increasing number of examinations in patients with previously implanted meshes for either repair-related problems or other indications [1]. For evaluation of these patients, it is important to identify the mesh itself separated from the soft tissues which surround it, and to confirm a successful reconstruction, identify mesh failure, or better plan a salvage repair [12].

LW mesh with the reduced polypropylene content and larger pore sizes between filaments have demonstrated a pronounced reduction in inflammatory reaction and improved integration into surrounding tissue in humans [13]. Based on the development tendency of minimal foreign body left behind, the heavyweight (HW) meshes are being gradually replaced by the LW meshes [14]. The previous studies showed that the LW mesh is not visible on CT because it is isoattenuating relative to surrounding tissues [1, 15].

**Fig. 1:**
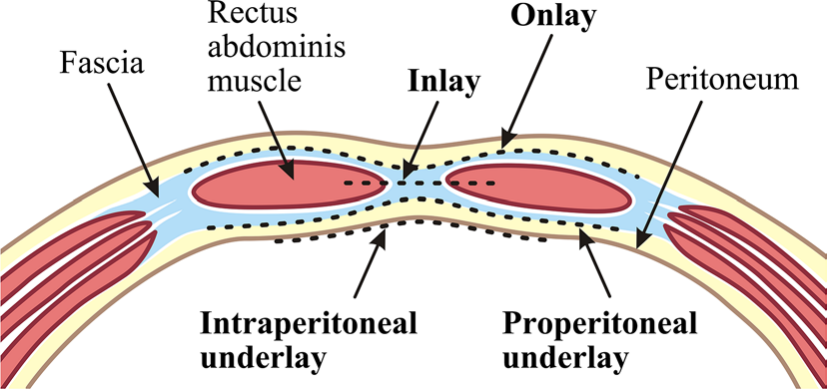
Anterior abdominal wall in cross section above the arcuate line. The mesh (black dashed lines) may be anterior to the abdominal wall fascia (Onlay), be adjacent to the fascial edges (Inlay), in the preperitoneal space between the fascia and the peritoneum (Properitoneal underlay), or be attached to the peritoneum in an intraperitoneal position (Intraperitoneal underlay)

Although the 2D HHUS has been shown to identify radiolucent foreign bodies [12, 16], our previous study showed that it has not been always reliable in identifying the LW mesh [17]. In ventral hernia repair surgery, mesh may be placed in a variety of locations in relation to the structures of the anterior abdominal wall (Fig. 1), all of which are usually close to the fascia [8, 11]. However, in sectional views (axial and sagittal views) of HHUS, both the LW mesh and fascia are usually rendered as a linear and hyperechoic area [17]. For this reason, the texture features of HHUS images reflect mixed properties of LW mesh overlapping fascia tissues. Fascia could be considered as anatomical noise to implanted LW mesh identification, and therefore reduce the differential diagnosis value of the computed texture features.

**Fig. 2:**
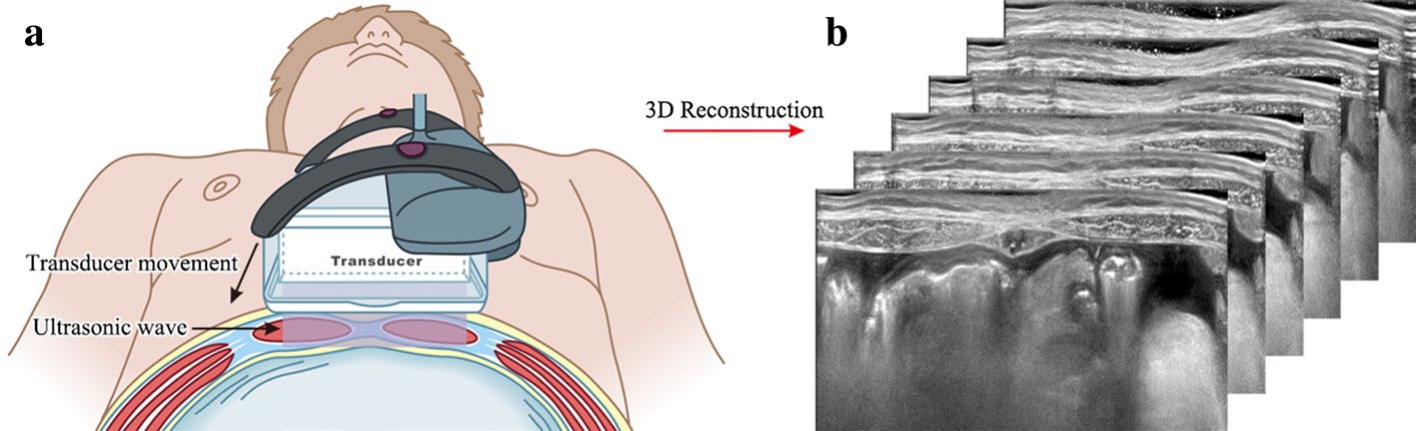
An illustrative example of **a** ABUS acquisition geometry with **b** the reconstructed tomographic anterior abdominal wall image

**Fig. 3:**
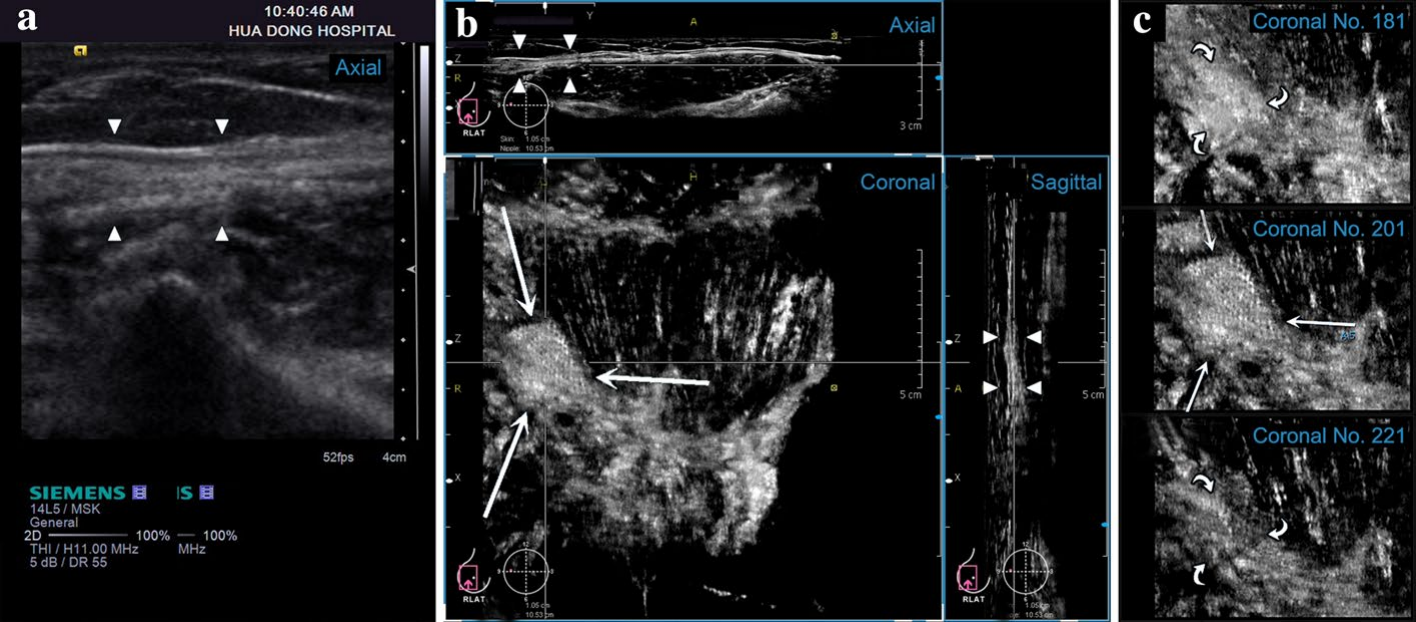
Differences of texture features in **a** the axial view of HHUS, **b** the three orthogonal views of ABUS and **c** coronal multislice views of ABUS for the same LW mesh. Note that the texture features of HHUS images reflect mixed properties of LW mesh overlapping fascia tissues. Therefore, it is difficult to identify the LW mesh itself separate from the fascia tissues that surround it (arrowheads). However, the new coronal plane of ABUS offers superior pore texture visualization, by separating the LW mesh (arrows) from the fascia (curved arrows) tissues

ABUS is an innovative new ultrasound imaging modality in which tomographic images of the scanning area are reconstructed in 3D from a series of 2D ultrasound images that are acquired by moving a conventional transducer (Fig. 2). Clinical trials have shown that ABUS provides superior tissue visualization and improved lesion conspicuity in comparison to HHUS, resulting in higher sensitivity and specificity [18–20]. As a relatively mature 3D ultrasound modality at present, the ABUS has drawn more and more attention from scholars [21]. It is not only widely applied in breast cancer detection, but also rapidly developed in abdominal wall hernias diagnosis [22, 23]. Compared to HHUS sectional views (Fig. 3a), ABUS also provides additional diagnostic information from coronal views, which cannot be generated by the 2D ultrasound [18]. The new coronal plane offers superior pore texture visualization, by separating the LW mesh from the fascia tissues (Fig. 3b, c). Therefore, ABUS could offer the ability to selectively analyze the texture features of the LW mesh, having the potential to provides more accurate features to characterize pore texture patterns, and ultimately provide more accurate measures for implanted LW mesh identification.

In this paper we present an exploratory study that investigates the potential advantages of ABUS pore texture analysis for implanted LW hernia mesh identification. In vitro, the appearances of four different flat meshes in both ABUS and HHUS images were evaluated and compared. In vivo, pore texture patterns of 87 hernia regions were analyzed both in ABUS images and their corresponding HHUS images. The presence of the previously implanted LW mesh in the hernia region was identified by using both ABUS and HHUS texture features. We compared the relative identification performance of ABUS and HHUS texture features in correlating with the established surgical findings. Although preliminary, our results suggest that ABUS pore texture analysis could potentially provide more discriminative features for implanted LW hernia mesh identification, in comparison to HHUS images. To the best of our knowledge, our study is the first to investigate the potential advantages of ABUS pore texture analysis for implanted LW hernia mesh identification, with the intention to offer instrumental evidence for the design of larger clinical studies in the future. The improved performance and low cost of ABUS will likely fuel the rapid and broad dissemination of ABUS as an implanted LW hernia mesh imaging modality.

## Results

### In vitro study

**Fig. 4:**
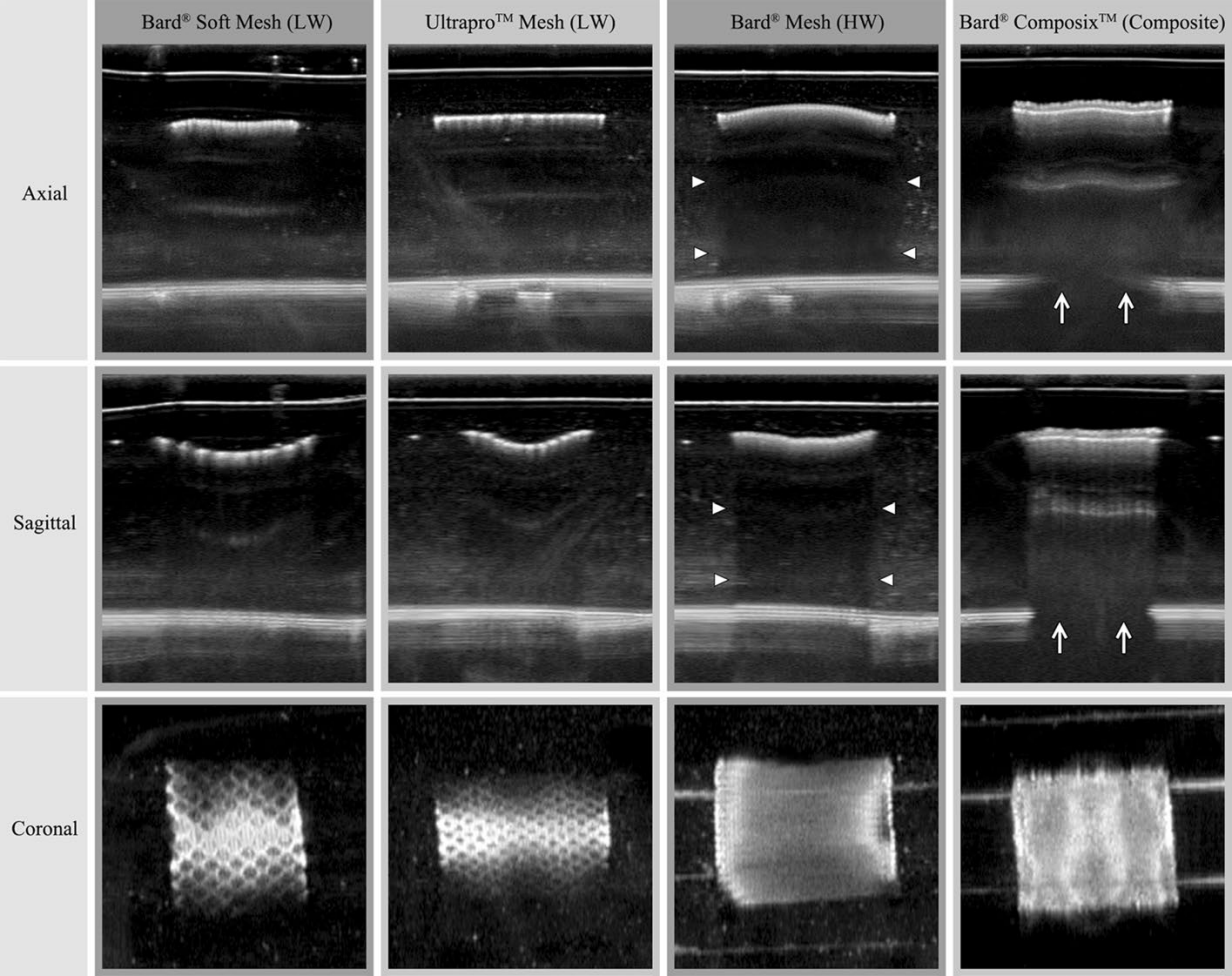
ABUS imaging comparisons of four tested meshes. The four columns show the Bard® Soft Mesh (LW), the Ultrapro™ Mesh (LW), the Bard® Mesh (HW), and the Bard® Composix™ Mesh (Composite) in three orthogonal views, respectively. Note that both LW Bard® Soft and LW Ultrapro™ meshes are shown in the coronal plane with distinct pore texture. The HW Bard® mesh is shown in sectional views (axial and sagittal views) with posterior acoustic shadowing (arrowheads). The Composite Bard® Composix™ mesh is shown in sectional views (axial and sagittal views) with badly posterior acoustic shadowing, and the bottom of the plastic container has been unable to observe by ultrasonography (arrows)

**Fig. 5:**
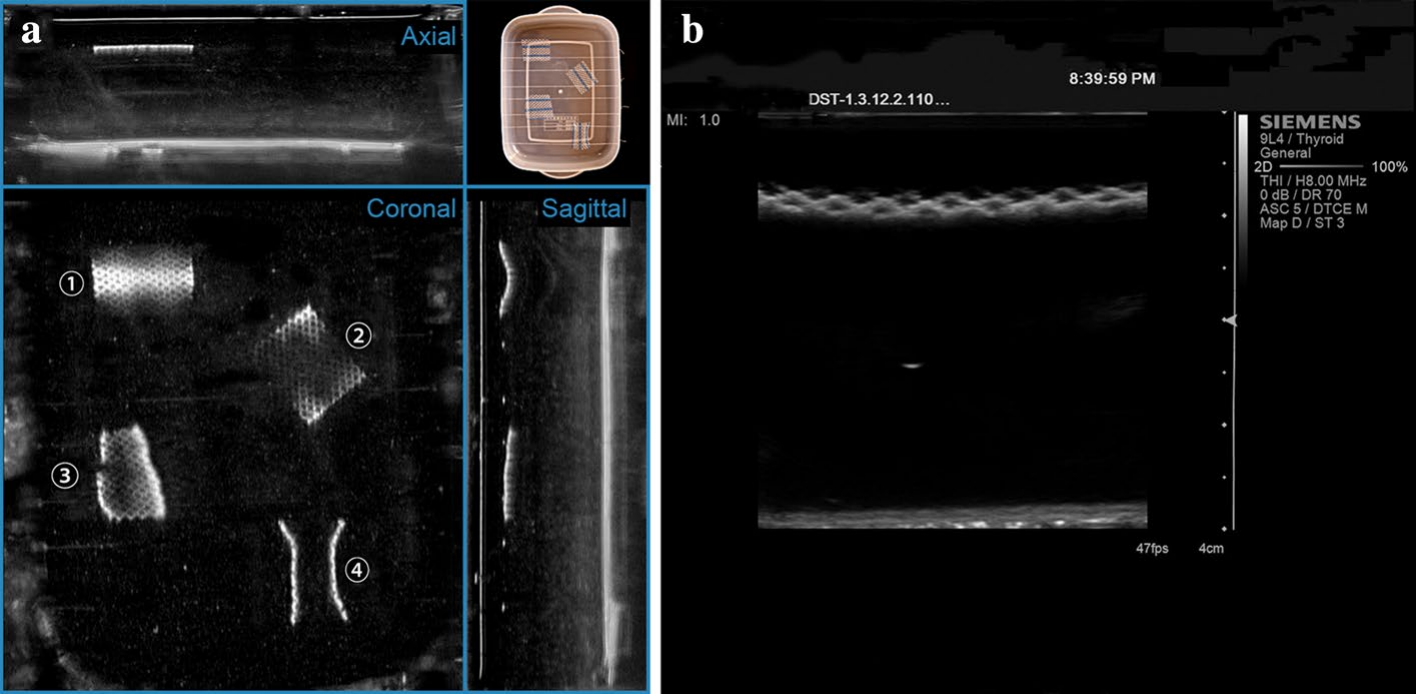
**a** Three orthogonal plan views of ABUS imaging of the Ultrapro™ Mesh box, while **b** is HHUS imaging result (axial plane). Note that fragment No. ➀ is parallel to the horizontal plane, fragment No. ➁ is rotated a certain angle in the horizontal plane, fragment No. ➂ is rotated a certain space angle in the depth direction of the horizontal plane, and fragment No. ➃ is curved to represent the shrinkage of the mesh. Note that the fragments No. ➀–➃—are used to simulate the mesh spatial variation that may occur after implantation.

**Fig. 6:**
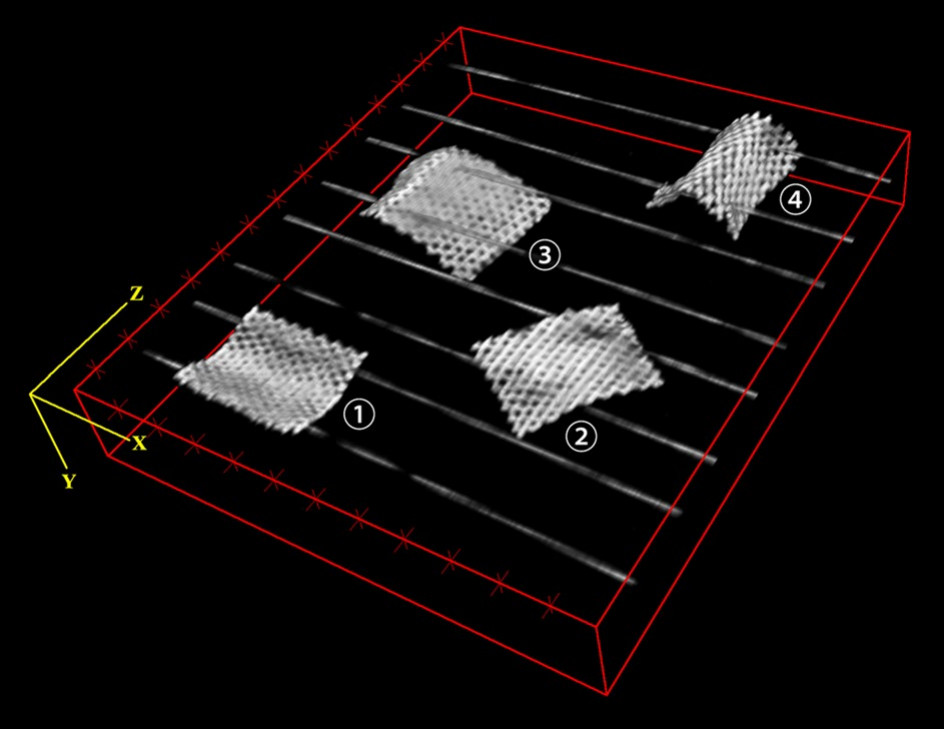
The 3D volume rendering results based on ABUS scan data of the Ultrapro™ Mesh box

**Fig. 7:**
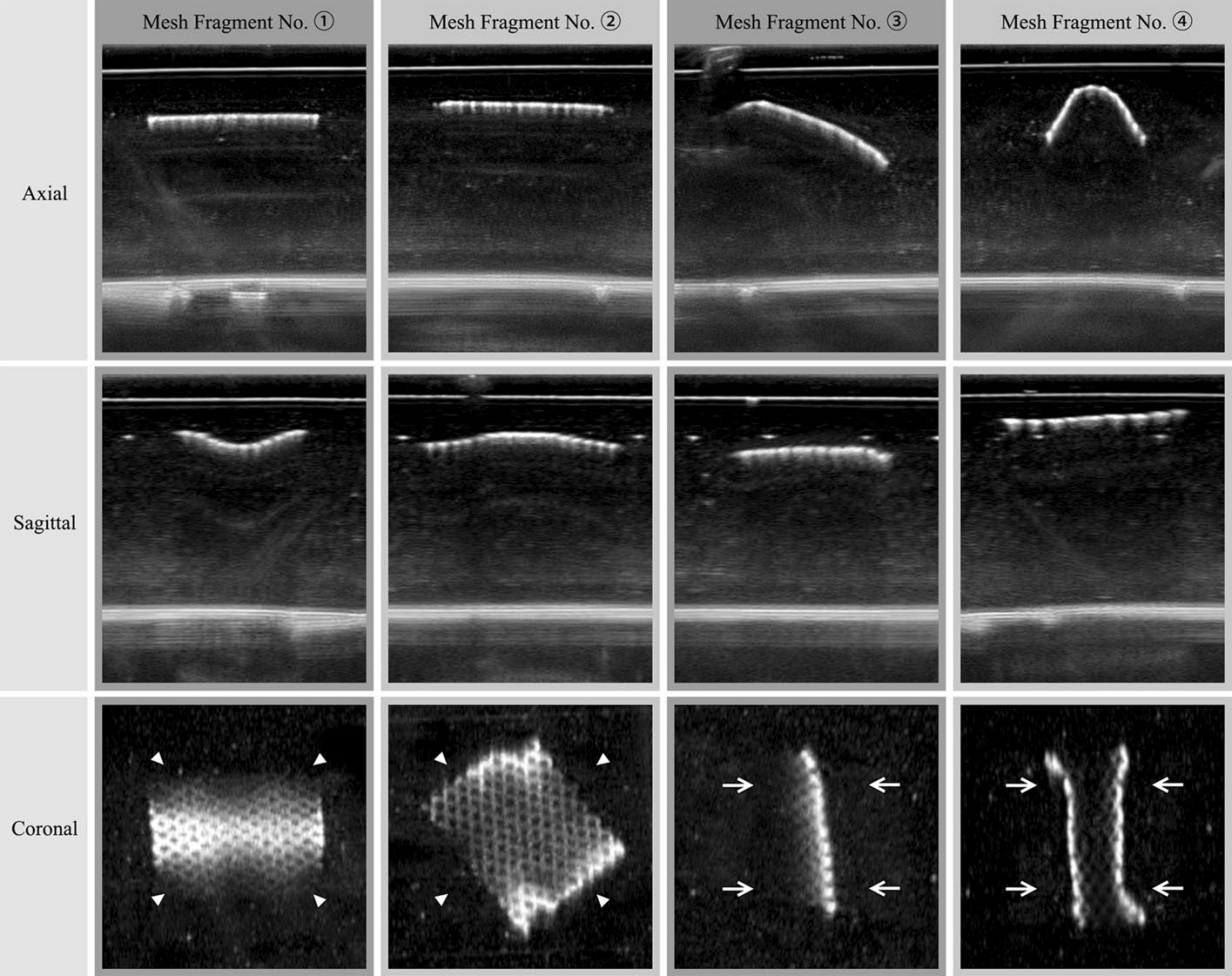
The results of ABUS imaging of Ultrapro™ Mesh (LW) with four mesh fragments. Note that fragment No. ➀ is parallel to the horizontal plane, fragment No. ➁ is rotated a certain angle in the horizontal plane, fragment No. ➂ is rotated a certain space angle in the depth direction of the horizontal plane, and fragment No. ➃ is curved to represent the shrinkage of the mesh. Note that the fragments No. ➀–➃—are used to simulate the mesh spatial variation that may occur after implantation

The ABUS imaging comparisons of four tested meshes are shown in Fig. 4. As an example, the three orthogonal plan views of ABUS imaging of the Ultrapro™ Mesh box are shown in Fig. 5a. In comparison, the HHUS imaging result (axial plans) is shown in Fig. 5b. The 3D volume rendering results based on ABUS scan data of the Ultrapro™ Mesh box are shown in Fig. 6, and the results of ABUS imaging of Ultrapro™ Mesh fragments No. 1 to No. 4 are shown in Fig. 7.

### In vivo study

**Fig. 8:**
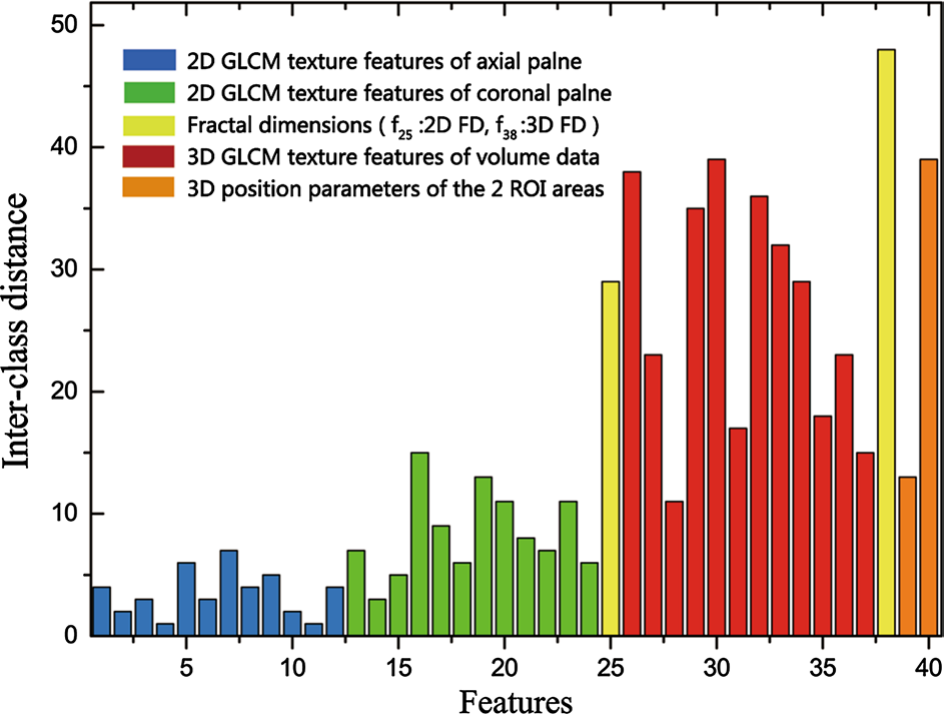
The inter-class distance results of all 40 pore texture features

The calculation results of the inter-class distance for all 40 pore texture features are shown in Fig. 8. The distinction rate between fascia and mesh is directly proportional to the parameter values of inter-class distance.

As shown in Fig. 8, the blue strip regions are 2D gray level co-occurrence matrix (GLCM) texture features of axial plane. The green strip regions are 2D GLCM texture features of coronal plane. The red strip regions are 3D GLCM texture features of volume data. There are 12 texture features in each of the blue, green and red regions. These features are energy ( *f*_1_), contrast ( *f*_2_), correlation ( *f*_3_), variance( *f*_4_), homogeneity ( *f*_5_), sum average ( *f*_6_), entropy ( *f*_7_), autocorrelation ( *f*_8_), dissimilarity ( *f*_9_), cluster shade ( *f*_10_), cluster prominence ( *f*_11_) and maximum probability ( *f*_12_) from left to right. The yellow strip regions are 2D and 3D fractal dimension (FD). And the orange strip regions are 3D position parameters of the 2 ROI areas.

**Fig. 9:**
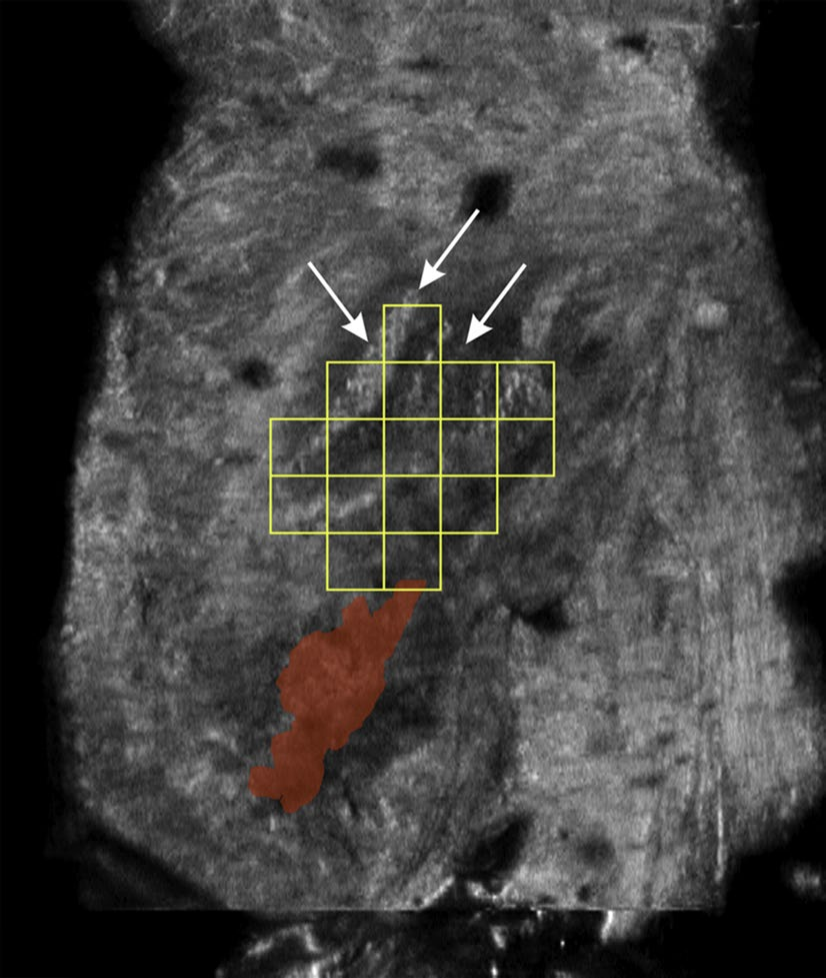
The LW mesh and hernia sac test results from an ABUS data

Incisional hernias often recur and form hernia sacs where the mesh was previously implanted. Therefore, the location of hernia sac is closely related to the location of incisional hernia and the location of mesh repair. Thus, the closer to the hernia sac, the higher occurrence of mesh. As an example, Fig. 9 shows the LW mesh and hernia sac test results from an ABUS data.

## Discussion

Precise visualization of meshes and their position would greatly aid in hernia recurrence risk assessment and the preoperative planning of salvage repair. However, since LW meshes are very thin thickness and large pore sizes between filaments, conventional 3D imaging techniques such as CT and MRI cannot visualize the LW meshes [1]. Patients sometimes have to undergo a second-look operation for visualizing the mesh implants [3]. An innovative new ABUS provides additional diagnostic information from coronal views, which cannot be generated by the 2D ultrasound [18]. The new coronal plane offers superior pore texture visualization, by separating the LW mesh from the fascia tissues. From Figs. 4, 5, 6 and 7, it can be seen that the imaging results of ABUS for implanted LW meshes are much more visualized and effective in comparison to HHUS. This is due to the high resolution of ABUS imaging, and its unique coronal plane provides additional diagnostic information for us.

HW meshes and composite meshes have large thickness and weight, small pore sizes between filaments or even no pore. For this reason, these two types of meshes can be observed by conventional 3D imaging methods (CT and MRI). While in ABUS condition, as shown in Fig. 4, the HW and composite meshes also can be observed by ABUS, the reason is that the conventional HW and composite mesh most commonly appears as a linear hyperechoic interface with posterior acoustic shadowing. This appear can provide the radiologist the ability to easily see the mesh in axial and sagittal views.

As shown in Fig. 5, the imaging result of HHUS for LW mesh is very similar to the axial and sagittal plane of ABUS. However, the scanning area of HHUS is very narrow, and the radiologist needs to manually integrate multiple images. While in ABUS condition, since the ABUS has a wide field of view, thus, it can display the entire abdominal wall area at once without missing any parts and makes the radiologist to perceive the implanted LW mesh easily.

From the Figs. 6 and 7, it can be seen that even if there is a spatial variation after the LW mesh implantation, the unique coronal view of ABUS imaging can be used to provide the spatial variation visualization of implanted LW mesh. As shown in Fig. 6 (The 3D volume rendering results based on ABUS scan data of the Ultrapro™ Mesh box), the ABUS can clearly observe the appearance of the implanted LW mesh after spatial variation.

In vivo study, Fig. 8 shows the inter-class distance results of all 40 pore texture features. From the blue, green and red strip regions in Fig. 8, we can see that the inter-class distance of 2D axial plane texture features (blue strip regions) are generally smaller than others. The reason is that the fascia and LW mesh are approximately presented as a linear bright strip in axial plane view, with little discrimination. Then, compared with the axial plane, the inter-class distance of 2D coronal plane texture features (green strip regions) are higher. The reason is that the LW mesh presents a unique mesh texture in the coronal plane view and offers superior pore texture visualization. However, due to the mesh may have spatial variation such as the curl and shrinkage after implantation, the contribution of 2D coronal plane texture features to LW mesh and fascia identification is not significant. Finally, the inter-class distance of 3D coronal plane texture features are greatly increased compared with 2D coronal plane. The reason is that the use of 3D pore texture features significantly improved the effectiveness and robustness of the identification method in distinguishing between LW mesh and fascia.

As shown in Fig. 8 (yellow strip regions), since the FD feature is more suitable for the analysis of the mesh texture, the inter-class distance of 2D FD and 3D FD features are higher than other inter-class distance of features. The inter-class distance of 3D FD feature reaches the maximum value in the inter-class distance of 40 features, which fully reflects that FD features can effectively distinguish LW meshes from fascia.

As shown in Fig. 8 (orange strip regions), firstly, although the four tension-free meshes placement positions were located at a scan depth of 1–3 cm and the fascia in this area has a higher probability of occurrence, the effect of feature *f*_*39*_ (local feature) based on scanning depth to LW mesh and fascia identification is unsatisfactory. Secondly, for recurrent cases of abdominal wall hernia, the repair location of the original abdominal wall hernia is the high incidence of recurrent hernia, thus, the closer to the hernia sac, the higher occurrence of mesh. Therefore, the feature *f*_*40*_ (environmental feature) based on the coronal plane position in the region of interest (ROI) can distinguish the LW mesh and the fascia significantly, and it’s inter-class distance ranks the third in the inter-class distance ordering of all 40 features.

The 25 features with the high distinction for fascia and mesh were selected according to the order of inter-class distance from large to small, in order to reduce the feature dimension of subsequent classification recognition, improve classification accuracy and reduce classification working time.

Our results provide compelling evidence that ABUS has the potential to provides more accurate features to characterize pore texture patterns. ABUS can provide a broader view which is more convenient for sonologists to observe the entire mesh at one time. Meanwhile, because ABUS can provide more intuitive and easier understood 3D information, sonologists do not need to reconstruct the spatial structure of 3D LW mesh manually in their mind.

Our study, nevertheless, has certain limitations. The small sample size does not provide sufficient statistical power to connote general applicability of our results and to fully determine the superiority of the 3D versus the 2D pore texture analysis methods. However, since this is the first study to explore the pore texture analysis in ABUS images for implanted LW hernia mesh identification, our intention was to evaluate proof-of-concept and demonstrate the instrumental evidence required to initiate the design of large clinical studies in the future. Such larger studies will render the sufficient statistical power required to fully evaluate the potential advantages of ABUS imaging in LW mesh identification.

## Conclusions

Although preliminary, our results suggest that the mesh pore texture provided by ABUS in the coronal plane is particularly effective in assessing the presence of implanted LW meshes. Compared to HHUS imaging, ABUS has the potential to provides more accurate features to characterize mesh pore texture patterns and ultimately provide more accurate measures for implanted LW mesh identification.

## Materials and methods

### In vitro study

**Fig. 10:**
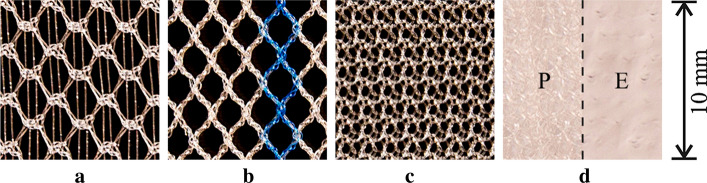
The top view of four tested meshes. Each photograph shows a 10 mm wide piece of the mesh material. **a** Bard® Soft Mesh (LW). **b** Ultrapro™ Mesh (LW). **c** Bard® Mesh (HW). **d** Bard® Composix™ Mesh (Composite). This composite mesh is constructed of polypropylene mesh on one side (P), bonded with expanded polytetrafluoroethylene (ePTFE) on the other (**e**)

**Fig. 11:**
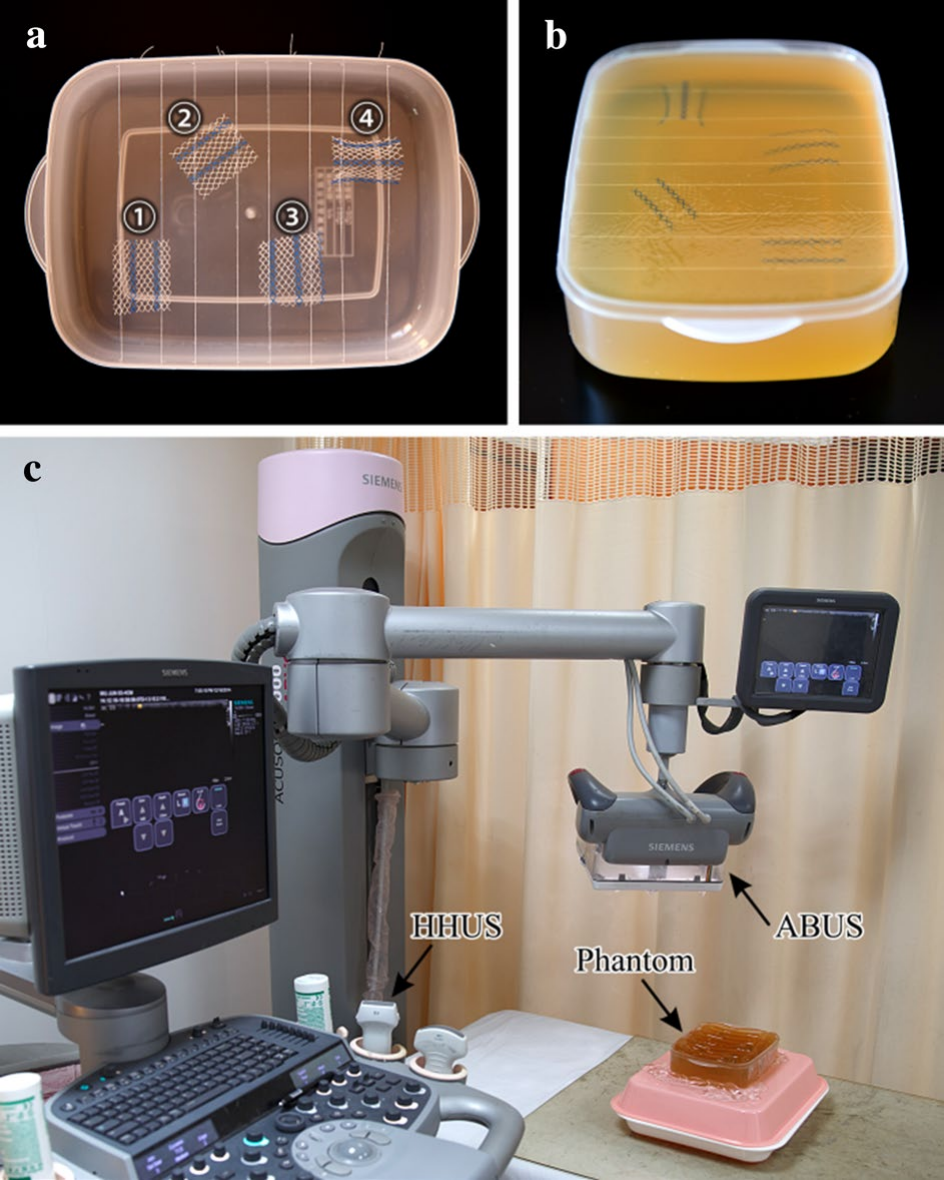
**a** Plastic container used to hold 4 fragments of the LW Ultrapro™ Mesh, while **b** shows the plastic container filled with gelatin. **c** The experimental equipment of in vitro study. Note that the fragments No. ➀–➃—are used to simulate the mesh spatial variation that may occur after implantation.

In vitro study, four different types of flat meshes (Fig. 10) were tested. Table 1 shows the basic descriptive data about the four tested meshes. Due to the mesh spatial variation may occur after implantation, 4 fragments (Fig. 11a, fragments No. ➀–➃) of Ultrapro™ Mesh (LW) were used to simulate the mesh spatial variation. First, cotton threads were used to control and fix mesh fragments gestures in a plastic container (Fig. 11a). Then, the plastic container filled with gelatin (Fig. 11b). Finally, HHUS and ABUS imaging were performed separately, and the imaging results were evaluated and compared by 2 experienced board-certified radiologists. It’s worth noting that the ultrasonic coupling agent was both used in HHUS and ABUS imaging. Figure 11c shows the experimental equipment of the in vitro study.

**Table 1:** Basic descriptive data about the four tested meshes

Brand name	Bard® Soft Mesh	Ultrapro™ Mesh	Bard® Mesh	Bard® Composix™ Mesh
Mesh type	Lightweight (LW)	LW	Heavyweight (HW)	Composite
Manufacturer	Bard Davol Inc	Ethicon, Johnson & Johnson	Bard Davol Inc	Bard Davol Inc
Material	Polypropylene	Polypropylene-poliglecaprone	Polypropylene	Polypropylene-ePTFE
Area weight (g/m^2^)	40	50	102	250
Thickness (mm)	0.5	0.5	1.0	2.0
Pore size (mm)	3.5	3	1	n/a
Maximum Pressure	n/a	525 mmHg	1650 mmHg	n/a
Instructions by manufacturer	LW mesh designed for open and laparoscopic hernia repair	Partially absorbable LW mesh for abdominal wall reinforcement	Conventional polypropylene HW mesh for strong repair	Composite mesh with clinically proven materials to mitigate the risk of visceral adhesion

### In vivo* study*

#### Study population

From December 2018 to September 2019, 87 patients (56 male and 31 females; age range, 43–79 years; mean age ± SD, 60.6 ± 11.2 years) were recruited for the in vivo study. The study was approved by the Ethics Committee of Huadong Hospital. All patients were volunteers who signed informed consent. The patients were referred to our ultrasound department for specific diagnostic queries, such as erythema, swelling, pain in the hernia region, intensified screening for symptoms suspicious for hernia recurrence, and preoperative diagnosis. All patients underwent HHUS and ABUS examinations in the hernia region. To obtain the evaluation criteria for confirming the LW mesh identified with ABUS and HHUS pore texture features, only the patients who subsequently underwent surgery were considered in our study. Based on the surgical findings, 38 of these patients (44%) with previously implanted LW hernia mesh constituted the case group, and 49 patients (56%) with no implanted LW hernia mesh constituted the control group. After anonymization, the HHUS and ABUS dataset was then available for further study.

### Examination methods (HHUS vs. ABUS)

The HHUS examinations were performed by a radiologist with five years’ experience in abdominal wall hernia sonography using an ACUSON S2000 ultrasound system (Siemens Medical Solutions, Mountain View, CA, USA). The patients were placed in the supine position and breathing normally for the examination. The Static 2D images were obtained using a 14L5 linear array transducer at 11 MHz ventral frequency. Images in axial and sagittal planes that best manifested the central hernia region were stored on an offline workstation for review and analysis.

The ABUS examinations were performed also on Siemens ACUSON S2000 by the same radiologist who with five years’ experience in abdominal wall hernia sonography and half a year in operating ABUS, but with a 14L5BV linear transducer. The central frequency of transducer varied from 9 to 11 MHz. The frequency was set at 11 MHz for the present study. The radiologist needs a great deal of experience in performing HHUS examinations. However, ABUS is an automated ultrasound examination. Depending on the shape, size and density of the abdominal cavity, the system automatically adjusts overall gain, frequency, focal zone placement depth and depth [23]. Therefore, ABUS examination requires less experience for radiologists than HHUS examination does. This transducer is able to capture a volume of 154 × 168 × 60 *mm*^*3*^ in a single scan. In each scanning, the ABUS generates 318 2D slices with a layer thickness of 0.5 mm in axial direction. ABUS examinations were performed with the patients lying in the same position and breathing normally as they were for the HHUS scanning. Before scanning, the radiologist applied appropriate pressure to the transducer to keep the probe closely attached to the central hernia region surface (Fig. 2a), therefore, the patient’s normal breathing can’t influence the results. Each scan takes 65 s for typical ABUS image of mesh. After the acquisition, the volume data sets were automatically sent from the ultrasound system to the diagnostic workstation, which enabled comprehensive analysis and manipulation of the 3D data.

### Image analysis

**Fig. 12:**
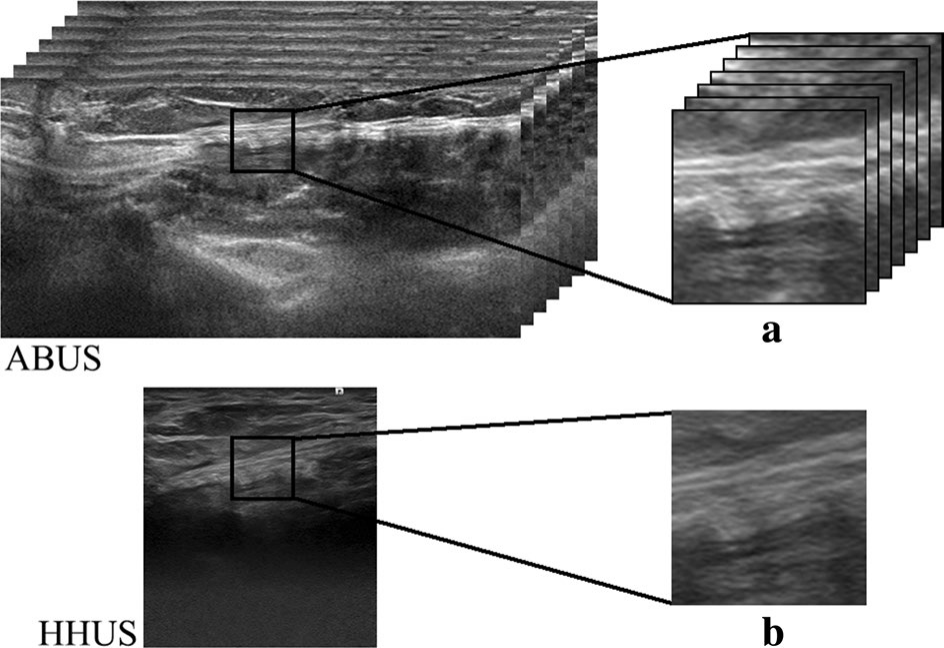
An illustrative example of **a** a 3D ROI segmented from a reconstructed automated 3-D breast ultrasound (ABUS) image and **b** the corresponding 2D ROI from the HHUS of the same central hernia region

A ROI was manually segmented from the central hernia region in each image. The physical dimensions of the ROIs were selected to be 10 mm^3^ for the ABUS images, and 10 mm^2^ for the HHUS images, based on previous suggestions for the average pore size of LW mesh in the literature [13]. Corresponding to these physical dimensions, 143 × 143 × 19 pixel ROIs at 0.07 mm/pixel in-plane resolution and 0.53 mm tomographic slice spacing were segmented from all the reconstructed ABUS images; matching 143 × 143 pixel ROIs at 0.07 mm/pixel resolution were segmented in the corresponding HHUS images of the same central hernia region. Examples of such ROIs are shown in Fig. 12.

To characterize the pore texture pattern, texture features of energy, contrast, correlation, variance, homogeneity, sum average, entropy, autocorrelation, dissimilarity, cluster shade, cluster prominence, maximum probability and FD were estimated from all the ABUS and HHUS ROIs. These texture features were originally defined for the analysis of 2D images. While texture analysis method has been widely implemented for the analysis of 2D medical images [24], the available techniques for 3D pore texture analysis are currently limited. Recently YAHIA Samah et al. demonstrated an efficient method for the analysis of textures in the 3-dimensional space [25]. In our study, two approaches were implemented for pore texture analysis in the 3D reconstructed ABUS images: tomographic (2D) and volumetric (3D) texture analysis [26].

### Tomographic (2D) texture analysis

The GLCM is one of the most known texture analysis operators. Based on measuring texture features, this operator computes the order of co-occurrence of pixels pairs at a certain direction and distance [25]. Haralick et al. defined GLCM [27]. We give a uniform definition of gray-level co-occurrence matrix for both 2D and 3D data. Consider an image (either 2D or 3D) which is rebound to *G* gray levels where *G* is a positive integer. The difference of spatial locations of two-pixel points (2D) or two voxels (3D) in an image can be described by a displacement vector *D*. For an image of *G* gray levels, the *G* × *G* GLCM *P*_*ij*_ for a displacement vector *D* is defined as follows. The entry (*i*, *j*) of *P*_*ij*_ is the number of occurrences of voxel-pair of gray levels *i* and *j* whose spatial locations are a vector *D* apart. When normalized by the total counts, the entry (*i*, *j*) of *P*_*ij*_, denoted as *P*(*i*, *j*), represents the probability of occurrence of voxel pair of gray levels *i* and *j* whose spatial locations are a vector *D* apart. In this definition, the co-occurrence matrix *P*_*ij*_ is a function of the displacement vector *D* which can be decomposed into a norm-1 distance *d* and a direction. Thus, *P*_*ij*_ describes distributions of certain spatial patterns of scale *d* in a certain direction [28].

**Fig. 13:**
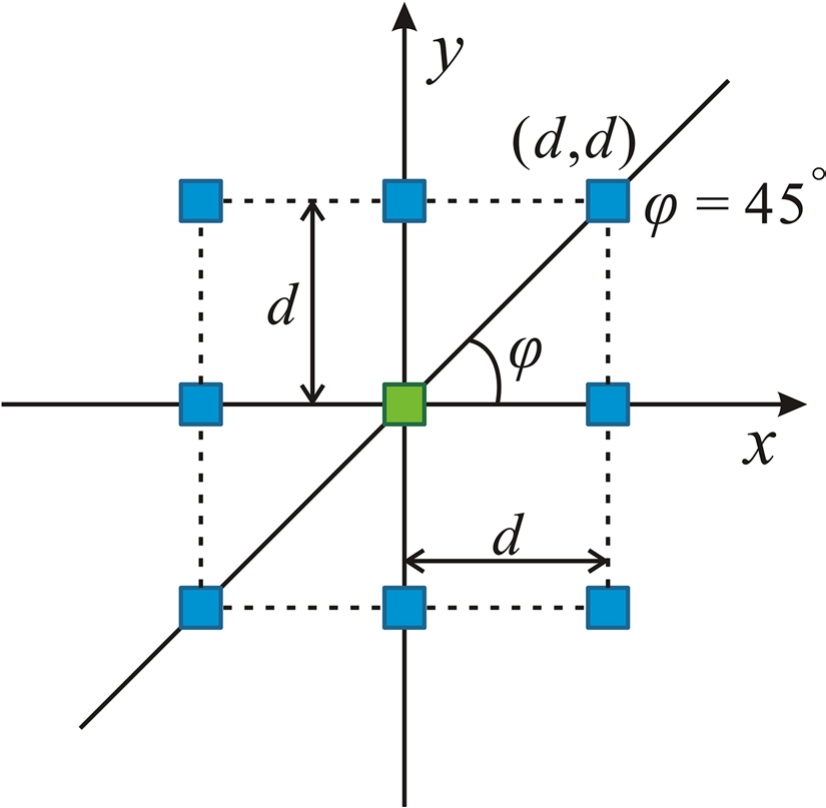
Spatial relations of a pair of pixels in 2D. For a particular pixel (green), it has eight neighboring pixels (blue) of norm-1 distance *d* in 4 independent directions

In 2D images, the difference of spatial locations of two pixel points can be described by displacement vector *D*(*φ*, *d*), where *φ* is the angle of two pixel points and the coordinate axis, and *d* is the distance between two pixel points. For 2D images, the possible spatial relations of a pair of pixels are shown in Fig. 13. Given a certain distance *d*, there are eight neighboring pixel pairs in four independent directions: horizontal, vertical, diagonal and anti-diagonal (corresponding to 0°, 90°, 45° and 135°, respectively).

Haralick et al. proposed 14 texture features, twelve of these features were selected in our study, including energy ( *f*_1_), contrast ( *f*_2_), correlation ( *f*_3_), variance( *f*_4_), homogeneity ( *f*_5_), sum average ( *f*_6_), entropy ( *f*_7_), autocorrelation ( *f*_8_), dissimilarity ( *f*_9_), cluster shade ( *f*_10_), cluster prominence ( *f*_11_), maximum probability ( *f*_12_). The co-occurrence frequencies were calculated symmetrically in the four independent directions around each pixel using a displacement vector *D* = (*dx*, *dy*) along *x* and *y* dimensions, where *dx* = *dy* = *1* pixel offset [26]. The texture features calculated in each of these four independent directions were averaged to create a single measure that was used in our experiments.

1 $$f_{1} = \sum\limits_{i} {\sum\limits_{j} {P(i,j)} }^{2}$$

2 $$f_{2} = \sum\limits_{n = 0}^{{N_{g} - 1}} {n^{2} \left\{ {\sum\limits_{i = 1}^{{N_{g} }} {\sum\limits_{j = 1}^{{N_{g} }} {P(i,j)\left\| {i - j} \right\| = n} } } \right\}}$$

3 $$f_{3} = \frac{{\sum\limits_{i} {\sum\limits_{j} {(i \cdot j)P(i,j) - \mu_{x} \mu_{y} } } }}{{\sigma_{x} \sigma_{y} }}$$

where ${\mu }_{x}$, ${\mu }_{y}$,${\sigma }_{x}$,$\mathrm{a}$ nd ${\sigma }_{y}$ are the average value and standard deviation of the rows and columns of matrix.

4 $$f_{4} = \sum\limits_{i} {\sum\limits_{j} {\left( {i - \mu } \right)^{2} P(i,j)} }$$

5 $$f_{5} = \sum\limits_{i} {\sum\limits_{j} {\frac{P(i,j)}{{1 + (i - j)^{2} }}} }$$

6 $$f_{6} = \sum\limits_{i = 2}^{{2N_{g} }} {iP_{x + y} (i)}$$

7 $$f_{7} = - \sum\limits_{i} {\sum\limits_{j} {P(i,j)\log (P(i,j))} }$$

8 $$f_{8} = \sum\limits_{i} {\sum\limits_{j} {(i \cdot j)P(i,j)} }$$

9 $$f_{9} = \sum\limits_{i} {\sum\limits_{j} {\left| {i - j} \right| \cdot P(i,j)} }$$

10 $$f{}_{10} = \sum\limits_{i} {\sum\limits_{j} {(i + j - \mu_{x} - \mu_{y} )^{3} P(i,j)} }$$

11 $$f_{11} = \sum\limits_{i} {\sum\limits_{j} {(i + j - \mu_{x} - \mu_{y} )^{4} P(i,j)} }$$

12 $$f_{12} = \mathop {MAX}\limits_{i,j} P(i,j)$$

where *N*_*g*_ is the maximum gray-level value and *P*(*i*, *j*) is the normalized GLCM. To optimize the computation of the gray-level co-occurrence statistics, gray-level quantization was implemented.

The average value and standard deviation of the rows and columns of matrix are:

13 $$\mu_{x} = \sum\limits_{i} {\sum\limits_{j} {iP(i,j)} }$$

14 $$\mu_{y} = \sum\limits_{i} {\sum\limits_{j} {jP(i,j)} }$$

15 $$\sigma_{x} = \sum\limits_{i} {\sum\limits_{j} {(i - \mu_{x} } } )^{2} P(i,j)$$

16 $$\sigma_{y} = \sum\limits_{i} {\sum\limits_{j} {(j - \mu_{y} )^{2} P(i,j)} }$$

FD was estimated based on the power spectrum of the Fourier transform of the image [26]. The 2D Discrete Fourier Transform (DFT) was performed using the Fast Fourier Transform (FFT) algorithm as:

17 $${\text{F(}}u{,}v{)} = \sum\limits_{{{\text{m}} = {0}}}^{{\text{M - 1}}} {\sum\limits_{{{\text{n}} = {0}}}^{{\text{N - 1}}} {\text{I(m,n)}} } e^{ - j(2\pi /M)um} e^{ - j(2\pi /N)vn} ,u = 0{,}1,...,M - 1;v = 0,1,...,N - 1$$

where *I* is the 2D image region of size (*M*, *N*), and *u* and *v* are the spatial frequencies in the *x* and *y* directions. The power spectral density *P* was estimated from *F*(*u*, *v*) as:

18 $$P(u,v) = \left| {F(u,v)} \right|^{2}$$

Finally, in order to compute the 2D FD, *P* was averaged along the radial slices direction across the FFT frequency domain. The frequency space was uniformly divided in 24 directions, with each direction uniformly sampled at 30 points along the radial component. The least-squares-fit of the *log*( *P*_*f*_) versus *log*( *f*) was estimated, where $f = \sqrt {u^{2} + v^{2} }$ denotes the radial frequency. The 2D FD is related to the slope *β* of the logarithmic curve in the following form:

19 $$FD = \frac{{3D_{T} + 2 - \beta }}{2} = \frac{8 - \beta }{2}$$

where *D*_*T*_ is the topological dimension, and is equal to *D*_*T*_ = 2 for a 2D image.

### Volumetric (3D) texture analysis

When calculating gray-level pore texture statistics, traditional 2D texture descriptors were extended to 3D by considering a 3D neighborhood of voxels (i.e., volume elements) instead of a 2D neighborhood of pixels.

**Fig. 14:**
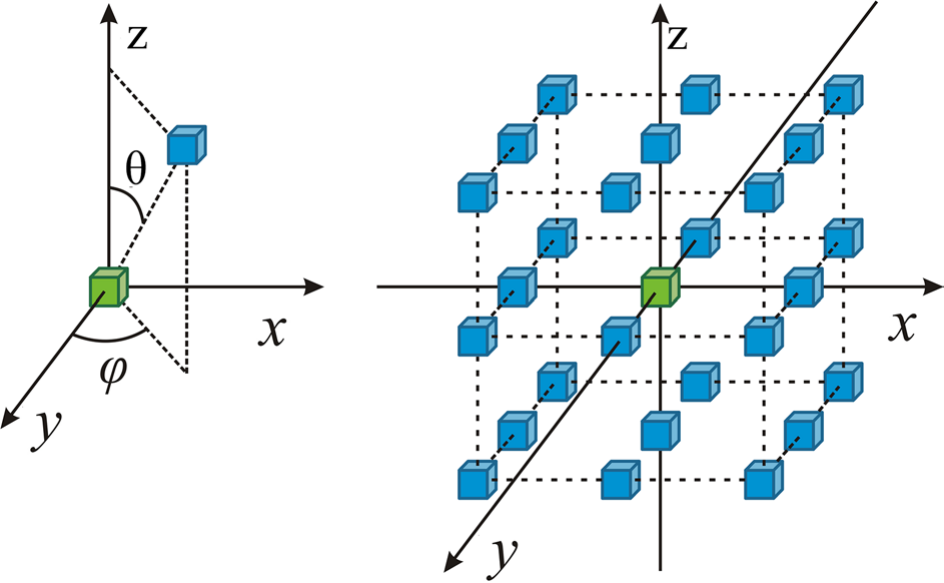
Spatial relations of a pair of voxels in 3D. For a particular voxel (green), it has 26 neighbors (blue) of norm-1 distance *d* in 13 independent directions

In 3D images, the difference of spatial locations of two voxels can be described by a displacement vector *D*(*φ*, *θ*, *d*), where *φ* and *θ* is the azimuth and the zenith angle respectively, and *d* is the distance between two voxels (Fig. 14 left). As shown in Fig. 14, there are totally 26 neighboring voxel-pairs in 13 independent directions. Here, it is still selected to extract the same 12 3D GLCM texture features as in 2D GLCM. The 3D displacement vector *D* = (*dx*, *dy*, *dz*) was defined around each voxel along the *x*, *y* and *z* dimensions, where *dx* = *dy* = *dz* = *1* is the voxel offset. Texture features were calculated in each of these 13 directions and they were averaged to create a single measure that was used in our experiments.

20 $$f_{1} = \sum\limits_{i} {\sum\limits_{j} {\sum\limits_{k} {P(i,j,k)} } }^{2}$$

21 $$f_{2} = \sum\limits_{i} {\sum\limits_{j} {\sum\limits_{k} {P(i,j,k)\left[ {(i - j)^{2} + (i - k)^{2} + (j - k)^{2} } \right]} } }$$

22 $$f_{3} = \sum\limits_{i} {\sum\limits_{j} {\sum\limits_{k} {P(i,j,k)\frac{{(i - \mu_{x} )(j - \mu_{y} )(k - \mu {}_{z})}}{{\sigma_{x} \sigma_{y} \sigma_{z} }}} } }$$

where ${\mu }_{x}$, ${\mu }_{y}$, ${\mu }_{z}$, ${\sigma }_{x}$, ${\sigma }_{y}$ and ${\sigma }_{z}$ are the average value and standard deviation of the rows and columns of matrix.

23 $$f_{4} = \sum\limits_{i} {\sum\limits_{j} {\sum\limits_{k} {\left| {P(i,j,k) - \sum\limits_{i} {\sum\limits_{j} {\sum\limits_{k} {P(i,j,k)} } } } \right|} } }^{2}$$

24 $$f_{5} = \sum\limits_{i} {\sum\limits_{j} {\sum\limits_{k} {\frac{P(i,j,k)}{{1 + \left[ {i - j)^{2} + (i - k)^{2} + (j - k)^{2} } \right]}}} } }$$

25 $$f_{6} = \sum\limits_{i = 2}^{{2N_{g} }} {iP_{x + y} (i)}$$

26 $$f_{7} = \sum\limits_{i} {\sum\limits_{j} {\sum\limits_{k} {P(i,j,k)\left[ { - \ln P(i,j,k)} \right]} } }$$

27 $$f_{8} = \sum\limits_{i} {\sum\limits_{j} {\sum\limits_{k} {(i \cdot j \cdot k)P(i,j,k)} } }$$

28 $$f_{9} = \sum\limits_{i} {\sum\limits_{j} {\sum\limits_{k} {\left| {i - j} \right| \cdot \left| {i - k} \right| \cdot \left| {j - k} \right| \cdot P(i,j,k)} } }$$

29 $$f{}_{10} = \sum\limits_{i} {\sum\limits_{j} {\sum\limits_{k} {(i + j + k - \mu_{x} - \mu_{y} - \mu_{z} )^{3} P(i,j,k)} } }$$

30 $$f_{11} = \sum\limits_{i} {\sum\limits_{j} {\sum\limits_{k} {(i + j + k - \mu_{x} - \mu_{y} - \mu_{z} )^{4} P(i,j,k)} } }$$

31 $$f_{12} = \mathop {MAX}\limits_{i,j,k} P(i,j,k)$$

where *P*(*i*, *j*, *k*) is the normalized GLCM. The average value and standard deviation of the matrix are:

32 $$\mu_{x} = \sum\limits_{i} {\sum\limits_{j} {\sum\limits_{k} {iP(i,j,k)} } }$$

33 $$\mu_{y} = \sum\limits_{i} {\sum\limits_{j} {\sum\limits_{k} {jP(i,j,k)} } }$$

34 $$\mu_{z} = \sum\limits_{i} {\sum\limits_{j} {\sum\limits_{k} {kP(i,j,k)} } }$$

35 $$\sigma_{x} = \sum\limits_{i} {\sum\limits_{j} {\sum\limits_{k} {(i - \mu_{x} )^{2} P(i,j,k)} } }$$

36 $$\sigma_{y} = \sum\limits_{i} {\sum\limits_{j} {\sum\limits_{k} {(j - \mu_{y} )^{2} P(i,j,k)} } }$$

37 $$\sigma_{z} = \sum\limits_{i} {\sum\limits_{j} {\sum\limits_{k} {(k - \mu_{z} )^{2} P(i,j,k)} } }$$

FD was estimated based on the power spectrum of the 3D Fourier transform of the image. FFT algorithm was used to perform 3D DFT for the entire 3D ROI area:

38 $$F(u,v,w) = \sum\limits_{m = 0}^{M - 1} {\sum\limits_{n = 0}^{N - 1} {\sum\limits_{k = 0}^{K - 1} {I(m,n,k)e^{ - j(2\pi /M)um} } } } e^{ - j(2\pi /N)vn} e^{ - j(2\pi /K)wk}$$

where *I* is the 3D image region of size (*M*, *N*, *K*), and *u*, *v* and *w* are the spatial frequencies in the *x*, *y* and *z* directions respectively. The power spectral density *P* was estimated as:

39 $$P(u,v,w) = \left| {F(u,v,w)} \right|^{2}$$

Finally, in order to calculate 3D FD, *P* was averaged along with the radial sector direction across the 3D FFT frequency domain. The frequency space was uniformly divided in 24 azimuth directions and 12 zenith angles, with each direction uniformly sampled at 30 points along with the radial component. The least-squares-fit of the *log*( *P*_*f*_) versus *log*( *f*) was estimated, where $f = \sqrt {u^{2} + v^{2} + w^{2} }$ represents the radial frequency, 3D FD was related to the slope *β* of the logarithmic curve in the following form:

40 $${\text{FD}}_{{{\text{3D}}}} = \frac{{3D_{T} + 2 - \beta }}{2} = \frac{11 - \beta }{2}$$

where *D*_*T*_ is the topological dimension and is equal to *D*_*T*_ = 3 for 3D image.

### Inter-class distance analysis

If we use a distance metric to measure the dissimilarity, we hope that a pattern is close to those in the same class but far away from different classes. Therefore, a good feature extractor should maximize the distances between classes after transformation. Inter-class distance is a feature selection process that classifies two types of images using one feature of the image each time. When a feature was used to distinguish two types of images, it is required not only to separate the two types of images without error, but also to minimize the intra-class distance and maximize the inter-class distance. A large inter-class distance indicates that patterns are close to each other if they are from the same class but are far from each other if they are from different classes. Thus, for our study, the larger the inter-class distance is, the better the effect of distinction for fascia and mesh is. We define the inter-class distance as:

41 $$d\left({C}_{i},{C}_{j}\right)=d\left({m}_{i},{m}_{j}\right)-s\left({C}_{i}\right)-s({C}_{j})$$

where ${m}_{i}$ and ${m}_{j}$ are the mean vectors of the class ${C}_{i}$ and the class ${C}_{j}$, respectively. And $s({C}_{i})$ and $s({C}_{j})$ is some measure of the scatter of the class ${C}_{i}$ and the class ${C}_{j}$, respectively.

### Pore texture analysis for implanted LW hernia mesh identification

As mentioned earlier, to characterize the pore texture pattern, texture features of energy ( *f*_1_), contrast ( *f*_2_), correlation ( *f*_3_), variance( *f*_4_), homogeneity ( *f*_5_), sum average ( *f*_6_), entropy ( *f*_7_), autocorrelation ( *f*_8_), dissimilarity ( *f*_9_), cluster shade ( *f*_10_), cluster prominence ( *f*_11_) and maximum probability ( *f*_12_) were estimated from all the ABUS and HHUS ROIs. These twelve texture features were extracted from 2D axial plane, 2D coronal plane and 3D coronal plane respectively. FD texture feature comes from 2 and 3D data respectively.

We performed pore texture analysis on the 2D ROIs of HHUS and 3D ROIs of ABUS. In all 38 cases (case group) with LW mesh in the incision area and 49 cases (control group) without mesh in the incision area, 278 typical sample areas with the same size as the area to be classified in this paper were selected manually by doctors. It includes of 125 typical areas with only LW mesh and 153 typical areas with only fascia. First, all 40 pore texture features that mentioned earlier were extracted for each ROI region. The extracted pore texture features include 25 2D features, 13 3D features, one local feature ( *f*_*39*_) based on the scanning depth of ROI region and one environmental feature ( *f*_*40*_) based on the relationship between ROI region and hernia sac location. Second, the 40 extracted features were used to calculate and normalize the feature parameters of all 278 sample areas. Then, the inter-class distance of each pore texture feature was calculated and sorted from large to small. Finally, the differences in inter-class distance were analyzed for selecting more discriminative features.

## Data Availability

The datasets used and/or analysed during the current study are available from the corresponding author on reasonable request.

## Abbreviations

2D, 3D: Two, three dimensional
ABUS: Automated 3D breast ultrasound
CT: Computed tomography
DFT: Discrete fourier transform
FD: Fractal dimension
FFT: Fast fourier transform
GLCM: Gray level co-occurrence matrix
HHUS: Hand-held ultrasound
HW: Heavyweight
LW: Lightweight
MRI: Magnetic resonance imaging
ROI: Region of interest
